# Efficacy of Glucagon-Like Peptide 1 (GLP-1) Receptor Agonists for Weight Loss Management in Non-Diabetic Patients

**DOI:** 10.7759/cureus.65050

**Published:** 2024-07-21

**Authors:** Ilma Vahora, Kiran Prasad Moparthi, Majdah T Al Rushaidi, Meghana Reddy Muddam, Omobolanle A Obajeun, Abdelrahman Abaza, Arturo P Jaramillo, Faten Sid Idris, Humna Anis Shaikh, Lubna Mohammed

**Affiliations:** 1 Internal Medicine, California Institute of Behavioral Neurosciences & Psychology, Fairfield, USA; 2 Internal Medicine, St. George's University School of Medicine, Chicago, USA; 3 General Practice, California Institute of Behavioral Neurosciences & Psychology, Fairfield, USA; 4 Psychology, California Institute of Behavioral Neurosciences & Psychology, Fairfield, USA; 5 Pediatrics, California Institute of Behavioral Neurosciences & Psychology, Fairfield, USA; 6 Pathology, California Institute of Behavioral Neurosciences & Psychology, Fairfield, USA

**Keywords:** diabetes, endocrinology, glucagon-like peptide receptor agonists, weight loss, obesity

## Abstract

The rising prevalence of obesity has led to a poor quality of life affecting millions worldwide. The lack of a healthy diet and exercise intervention are the major risk factors leading to obesity, as well as genetics. Obesity can lead to type 2 diabetes mellitus. However, there are many people who are obese and do not have an established diagnosis of diabetes but want to reduce their body weight to improve their quality of life. This review aims to discuss the efficacy of the diabetic pharmacologic agents, glucagon-like peptide-1 (GLP-1) receptor agonists, on body weight. The review follows the Preferred Reporting Items for Systematic Reviews and Meta-Analyses (PRISMA) guidelines 2020 and includes a comprehensive search strategy. The articles gathered are from the last five to 10 years. The articles are collected from distinguished databases such as PubMed, Google Scholar, ResearchGate, and Science Direct. Of the 698 studies identified based on the screening methods, 22 were assessed for eligibility and 10 studies were included in the final review. The findings of this systematic review provide a bigger picture of the efficacy and safety of glucagon-like peptide receptor agonist agents. The review thoroughly discusses the risk factors for obesity and provides a treatment strategy that can be utilized in clinical practice in the future. The review concludes that glucagon-like peptide agents act as pharmacologic treatments for reduction in body weight and also serve as cardioprotective agents.

## Introduction and background

Obesity remains a challenging aspect of medicine because prevention is simple but not easy. The prevalence of obesity in the United States as of 2023 is 41.9% and 39% globally [1]. Studies have shown that every two in five adults have obesity in the United States [1]. Obesity is at an incline due to the vast availability of high-caloric foods as well as adaptation to sedentary lifestyles [2].

According to the Centers for Disease Control and Prevention (CDC), more than 60% of American adults live an unhealthy and sedentary lifestyle [1]. Furthermore, 25% of Americans do not engage in any physical activity at all. As a result of being overweight or obese, 2.8 million adults die each year. Indirectly, obesity is one of the most important causes of heart disease, which represents the main cause of death in the United States [3]. Obesity directly does not cause mortality, but the complications usually associated with it have the utmost impact. 

Obesity is a risk factor for many diseases. It leads to type 2 diabetes mellitus, hypertension, coronary artery disease, and gallbladder disease [3]. Obesity is also a risk factor for other complications such as sleep apnea and osteoarthritis [4]. For the purpose of this research, the focus here will be diabetes mellitus. Diabetes mellitus is caused by a lack of insulin secretion, which is known as type I diabetes. Type II diabetes is caused by defective insulin secretion and the reduced sensitivity of receptors on the cells to respond to insulin [4]. The complications related to diabetes can be divided into two groups, macrovascular and microvascular. The macrovascular complications consist of ischemic heart disease, peripheral vascular disease, and cerebrovascular disease [4]. On the contrary, the microvascular complications include retinopathy, nephropathy, and neuropathy. Without proper management, these complications can lead to mortality [4].

The first line of medical treatment for diabetes mellitus after lifestyle changes is metformin. It exerts its effects by altering glucose metabolism via a reduction in gluconeogenesis and opposing the counteractive hormone, glucagon [5]. It also enhances insulin sensitivity in the tissues, making it an optimal choice for treatment. Another group of medications for diabetes mellitus include glucagon-like peptide-1 (GLP-1) receptor agonists [5]. The glucagon-like 1 receptor agonists also exist to help regulate and monitor glucose levels in the bloodstream. The glucagon-like receptor agonist serves as a hormone that is glucose-dependent and has many functions. It counteracts glucagon, which is a hormone that increases gluconeogenesis when the body is hypoglycemic. Glucagon-like peptide (GLP) has many metabolic effects such as the release of insulin (glucose-dependent), reduction in gastric emptying, decreasing inflammation, and augmenting diuresis and natriuresis [5]. Therefore, these attributes of the hormone make it efficient for use in individuals with type II diabetes mellitus as well as obesity in non-diabetics. Some examples that have been successful for weight loss in these cohorts are semaglutide, liraglutide, dulaglutide, exenatide, albiglutide, and lixisenatide. However, the dosage and type of medications vary and may result in different outcomes.

## Review

Methods

This systematic review followed the Preferred Reporting Items for Systematic Reviews and Meta-analyses (PRISMA) 2020 guidelines. A search study strategy was conducted with the aid of reputable databases such as PubMed, ResearchGate, Science Direct, and Google Scholar. A MeSH strategy was applied to narrow the search to the articles of relevance. The search was initiated using the keywords “weight loss”, “anti-obesity agents”, “glucagon-like receptor agonists”, and “semaglutide” and was combined using the Booleans “OR” and “AND.” Table 1 demonstrates a brief overview of the databases examined for article compilations as well as the search strategy utilized.

**Table 1:** Databases and search strategies used to collect articles

Search Strategy	Database	Number of Articles Identified
Obesity and glucagon-like peptide 1 agonist	Google Scholar	142
(("Obesity"[Mesh]) AND ("Obesity/diagnosis"[Mesh] OR "Obesity/drug therapy"[Mesh] OR "Obesity/etiology"[Mesh] OR "Obesity/genetics"[Mesh] OR "Obesity/pathology"[Mesh] OR "Obesity/physiopathology"[Mesh] )) AND ("Glucagon-like peptide-1 receptor/administration and dosage"[Mesh] OR "Glucagon-like peptide-1 receptor/agonists"[Mesh] OR "Glucagon-like peptide-1 receptor/classification"[Mesh] OR "Glucagon-like peptide-1 receptor/drug effects"[Mesh] OR "Glucagon-like peptide-1 receptor/physiology"[Mesh] OR "Glucagon-like peptide-1 receptor/therapeutic use"[Mesh] )	PubMed	271
Obesity and glucagon-like peptide 1 agonist	Science Direct	109
Obesity and glucagon-like peptide 1 agonist	Research Gate	76

Inclusion Criteria

The studies were selected for inclusion based on the following participant, intervention, and outcome parameters: patients with a body mass index (BMI) greater than 27 and patients who were non-diabetic and received glucagon-like receptor agonists on a weekly basis. Furthermore, the studies with the following criteria were included: studies written and published in English, focusing on both males and females of all ethnicities, involving only human participants and no animals, full-text articles, and articles published within the past 10 years (2013-2023).

Exclusion Criteria

The studies were restricted to exclude the following: animal participants, publications before 2013, patients with a BMI less than 27, publications in any other language than English, gray literature, book chapters, or conference papers.

**Figure 1:**
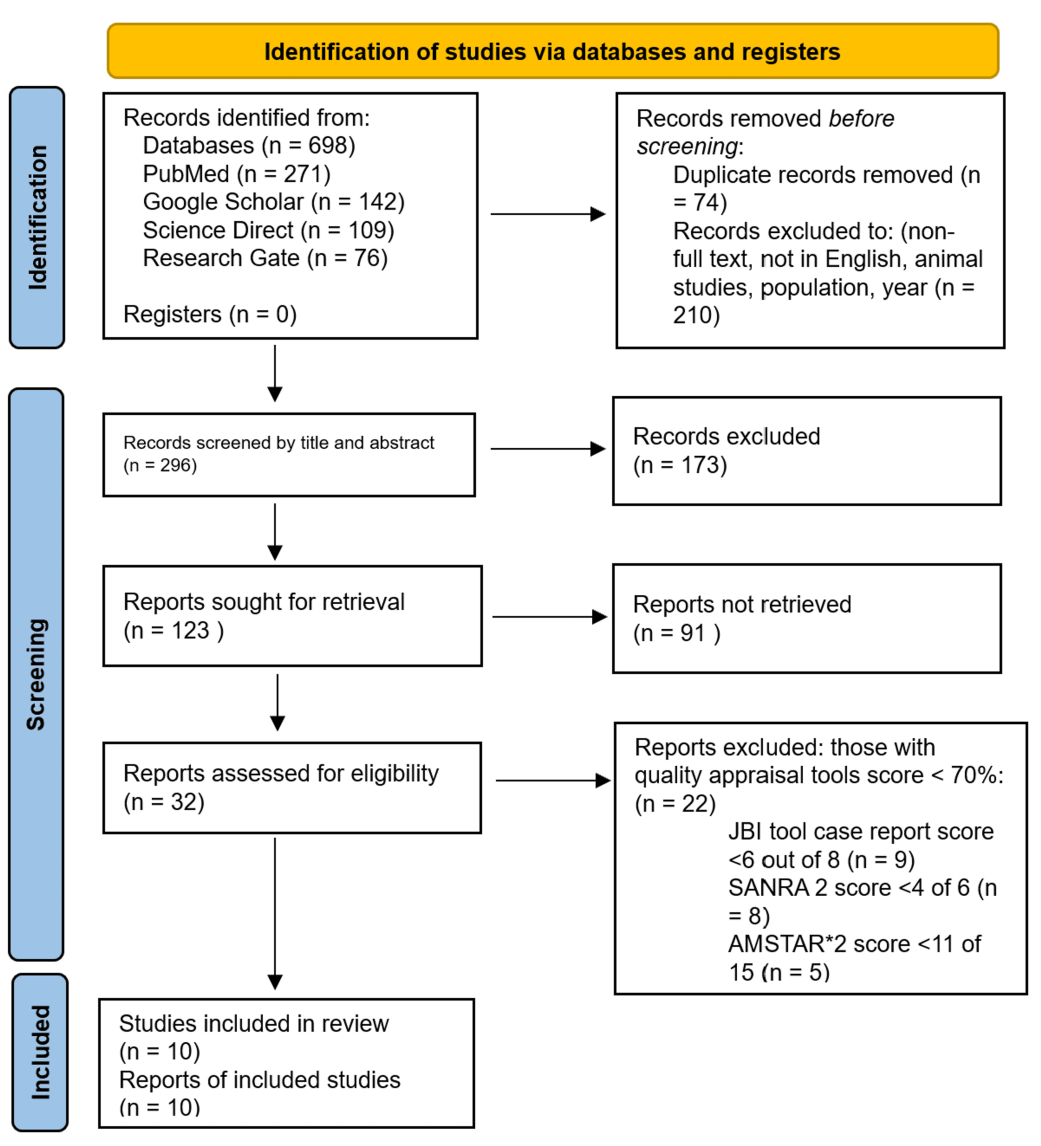
The PRISMA chart showing an overview of the screening process AMSTAR 2: Assessment of Multiple Systematic Reviews 2; SANRA: Scale for the Quality Assessment of Narrative Review Articles; JBI: Joanna Briggs Institute; PRISMA: Preferred Reporting Items for Systematic Reviews and Meta-Analyses

Figure 1 outlines the PRISMA chart, which gives an overview of the screening process [6].

Results

Using reputable databases and search strategies, articles were extracted and 11 were identified for the final review. These articles investigated the use of glucagon-like receptor agonists in patients with a BMI over 30.

Table 2 illustrates the articles collected from the databases, the study designs, the population involved in the studies, and the key findings of each study.

**Table 2:** Key findings related to the selected studies in this systematic review

Study	Study Design	Participants	Key Findings
Wilding et al. [1]	Double-blind trial	1,961 non-diabetic individuals with a body mass index ≥30.	In participants with overweight or obesity, 2.4 mg of semaglutide once weekly plus lifestyle intervention was associated with a persistent reduction in body weight [1].
Tan et al. [2]	Randomized controlled trial	3,613 individuals with obesity without diagnosed diabetes.	Obese individuals without an established diagnosis of type 2 diabetes mellitus showed an 11.85% reduction in weight loss with semaglutide compared to placebo. However, the risk of gastrointestinal adverse events, discontinuation of treatment, and serious adverse events were higher in the semaglutide group versus placebo.
Rubino et al. [3]	Randomized controlled trial	338 participants with an average age of 49 years; 265 women [78.4%]; mean [SD] body weight, 104.5 [23.8] kg; mean [SD] body mass index, 37.5. Participants were randomized (3:1:3:1) to receive once-weekly subcutaneous semaglutide, 2.4 mg or matching placebo, or once-daily subcutaneous liraglutide, 3.0 mg or matching placebo, plus diet and physical activity for a total of 68 weeks.	The average weight change was 15.8% with semaglutide and 6.4% with liraglutide [3]. Participants had significantly greater odds of achieving 10% or more, 15% or more, and 20% or more weight loss with semaglutide vs liraglutide (70.9% of participants vs 25.6% [odds ratio, 6.3 {95% CI, 3.5 to 11.2}], 55.6% vs 12.0% [odds ratio, 7.9 {95% CI, 4.1 to 15.4}], and 38.5% vs 6.0% [odds ratio, 8.2 {95% CI, 3.5 to 19.1}], respectively; all P<0.001). 13.5% of the participants receiving semaglutide discontinued treatment vs 27.6% with liraglutide. Gastrointestinal adverse events were reported by 84.1% with semaglutide and 82.7% with liraglutide.
O’Neil et al. [4]	Randomized, double-blind controlled trial	957 participants ≥18 years without diabetes and a body mass index of 30 or greater were randomly assigned to each active treatment in a 1:1 ratio, i.e., semaglutide 0.05 mg, 0.1 mg, 0.2 mg, 0.3 mg, or 0.4 mg; initiated at 0.05 mg per day and incrementally escalated every four weeks delivered once daily via subcutaneous injections.	Estimated mean weight loss was -2.3% for the placebo group versus -6.0% (0.05 mg), -8.6% (0.1 mg), -11.6% (0.2 mg), -11.2% (0.3 mg), and -13.8% (0.4 mg) for the semaglutide groups.
Garvey et al. [5]	Randomized controlled trial	304 participants with a mean body mass index of 38 were randomly assigned to semaglutide 2.4 mg (n=152) or placebo (n=152).	The mean change in body weight from baseline to week 104 was -15.2% in the semaglutide group (n=152) versus -2.6% with placebo (n=152), for an estimated treatment difference of -12.6 %-points (95% confidence interval, -15.3 to -9.8; P<0.0001) [5]. More participants in the semaglutide group than in the placebo group achieved weight loss ≥5% from baseline at week 104 (77.1% versus 34.4%; P<0.0001). Gastrointestinal adverse events, mostly mild-to-moderate, were reported more often with semaglutide than with placebo (82.2% versus 53.9%). In summary, in adults with overweight (with at least one weight-related comorbidity) or obesity, semaglutide treatment led to substantial, sustained weight loss over 104 weeks versus placebo.
Blundell et al. [6]	Double-blind randomized controlled trial	30 participants with obesity received 12 weeks of treatment with once-weekly subcutaneous semaglutide, dose-escalated to 1.0 mg.	After 12 weeks of treatment, the required energy intake was significantly reduced with semaglutide vs placebo accompanying weight loss with the use of semaglutide [6]. In addition to reduced energy intake, likely mechanisms for semaglutide-induced weight loss included less appetite and food cravings, better control of eating, and lower relative preference for fatty, energy-dense foods.
Ryan et al. [7]	Prospective cohort study	17,500 patients with a body mass index greater than 27 receiving 2.4 semaglutide weekly were followed for five years.	In a dose-ranging phase 2 study, a clinically relevant weight reduction was achieved with the use of semaglutide in patients with obesity who did not have a corresponding diagnosis of diabetes mellitus. The average weight reduction ranged from 6.0% to 13.8% from the baseline weight.
Ghusn et al. [8]	Retrospective cohort study	175 patients with a mean age of 49.3 and a mean body mass index of 41.3 received 1.7-2.4 mg semaglutide weekly for a duration of six months.	After a duration of three months, the average weight loss in the participant was 4.4 kg equivalent to 5.9% compared to a placebo of 3.7%. At six months, the weight loss reduction was 10.9% in participants taking semaglutide vs placebo (5.8%). Of the 102 patients who were followed up at six months, 89 (87.3%) completed weight loss of 5% or more, 56 (54.9%) accomplished weight loss of 10% or more, 24 (23.5%) achieved weight loss of 15% or more, and eight (7.8%) gained a weight loss of 20% or more.
Guo et al. [9]	Randomized controlled trial	5,867 without diabetes and overweight/obese received 2.4 mg weekly semaglutide.	The results showed that the treatment of overweight/obese patients without diabetes with glucagon-like 1 peptide agonists including liraglutide, exenatide, and semaglutide significantly achieved greater weight loss than placebo (-6.82 vs -3.96) and metformin (-5.87, -5.05). The analysis demonstrated that semaglutide displayed the most obvious anti-obesity effect with respect to weight loss, the reduction of body mass index, and waist circumference.
Friedrichsen et al. [10]	Double-blind randomized controlled trial	72 adults with obesity were randomized to once-weekly subcutaneous semaglutide (dose-escalated to 2.4 mg) or placebo for 20 weeks.	There was an observed suppression of appetite, improved eating control, reduction in cravings, and decreased required energy intake and body weight in participants taking 2.4 mg semaglutide weekly vs placebo.

Discussion

Obesity is a multifactorial disease that can occur at any age, irrespective of sex, geographic location, or socioeconomic status [6]. The worldwide prevalence of obesity continues to trend due to the increased intake of calories and a sedentary lifestyle. According to the World Health Organization (WHO), overweight and obesity are defined as an excessive accumulation of fat in the body that leads to health risks [7,8]. The BMI is an indicator of overweight and obesity. It is calculated by dividing the body weight in kilograms by the height, in meters [9,10]. The CDC and WHO define a normal BMI in the range of 18.5 to 24.9 kg/m^2^. However, a BMI over 25 kg/m^2^ is considered to be overweight and a BMI over 30 kg/m^2^ is defined as obese. Severe obesity would be deemed in individuals with a BMI over 40 kg/m^2 ^[11]. Obesity can’t be solely defined by numbers since it is a complex disease with various complications. The process of obesity does not occur overnight but over a long period of time, from months to years, depending on caloric intake. When the dietary energy input from excessive consumption of calories exceeds energy expenditure, obesity occurs [12]. When the dietary energy input is increased, it has to be stored somewhere. Therefore, it is converted to triglycerides and deposited in adipose tissue, which eventually causes weight gain when it expands [13]. Along with a lack of physical activity, the vast availability of affordable processed foods, increasing fast-food franchises, and health illiteracy have driven the trend of obesity worldwide [14]. Genetics and gut microbiome also play a role in developing obesity in a population. For example, Asians tend to contain about 2-5% more total body fat as compared to Caucasians [15,16].

Obesity can lead to many severe complications such as diabetes mellitus, obstructive sleep apnea, coronary artery disease, stroke, gallbladder disease, and many more devastating illnesses [17,18]. For the purposes of this review, the aim is to focus primarily on diabetes mellitus, caused by obesity. Type 2 diabetes mellitus is a common metabolic disorder amongst the population worldwide [19,20]. The pathophysiology of diabetes mellitus is driven by two mechanisms: the reduced insulin secretion by the β-cells of the pancreas and the insulin resistance by the insulin-sensitive tissues in the body, such as the muscles, liver, and adipose cells [21,22]. A malfunction in any one of these processes causes a metabolic imbalance and leads to the illness [23]. In the early stages of the disease, the reduced insulin sensitivity leads to a compensatory increased production of insulin by the pancreatic β-cells [24]. This accelerates the function of the β-cells in order to maintain normoglycemic levels. The aim of the mechanism is to prevent hyperglycemia by inducing hyperinsulinemia [25]. However, as time progresses, the pancreatic β-cells can’t compensate for the insulin resistance and decline in function [25,26]. The cells are exhausted and start to hypotrophy and die off [26]. In regards to obesity, it is hypothetically reasoned that insulin resistance seems to develop due to inflammation caused by the release of free fatty acids during lipolysis [27].

This review focuses on the population without an established diagnosis of type 2 diabetes mellitus. For comparison purposes, the criteria to be diagnosed with diabetes are discussed.

Type 2 diabetes mellitus is diagnosed by utilizing the glycated hemoglobin (A1C) test. This test measures the average glucose levels in the blood for the past three months. Results are interpreted as below [28].

Ø Below 5.7% is normal.

Ø Between 5.7% to 6.4% are diagnosed as pre-diabetes.

Ø 6.5% or higher on two separate readings is indicative of diabetes.

Furthermore, a fasting plasma glucose level of 126 mg/dL or greater is indicative of diabetes along with a random plasma glucose reading of 200 mg/dL or greater. A two-hour 75 g glucose tolerance test with a blood glucose level of 200 mg/dL or greater fits the criteria to establish diabetes as well [29].

Treatment

Various medications exist to maintain glucose levels in diabetics. First, metformin has been an essential treatment for decades to decrease blood glucose levels through its unique mechanisms of action. Its functions are complex and are not fully understood. At the molecular level, it has been theoretically demonstrated that metformin decreases insulin resistance by activating the enzyme adenosine monophosphate kinase (AMPK), which results in the inhibition of the enzymes that are involved in and regulate gluconeogenesis [29]. Metformin decreases glycogenesis in the liver and stimulates insulin signaling in the muscle cells thereby being known as an “insulin sensitizer” since the effects of metformin are not dependent on the pancreatic cell production of insulin [29]. The side effects of metformin include lactic acidosis, nausea, vomiting, and metallic taste in the mouth [30].

The other group of medications that are prescribed for diabetics and pertain to this review is GLP-1 receptor agonists. These include semaglutide, exenatide, lixisenatide, liraglutide, albiglutide, and dulaglutide [31]. These groups of medications are now indicated in diabetic patients who have a comorbid disease such as heart failure or chronic kidney disease [31]. These are also indicated in patients taking metformin but have not reached their target hemoglobin A1C goal in three months or in patients with a hemoglobin A1C greater than 1.5% over their goal. Not only have these agents shown to be beneficial to diabetics, but they also help weight loss in non-diabetic patients. Semaglutide and liraglutide are specifically Food and Drug Administration (FDA)-approved pharmacologic treatments that aid in weight reduction in non-diabetics. These medications are administered subcutaneously. When semaglutide is prescribed for diabetes, it is under the brand name Ozempic®. However, when it is prescribed for weight loss only, it is referred to as Wegovy®. Studies have shown that these medications show a significant reduction in weight and reduce cravings [31].

GLP-1 is a hormone found to lower blood glucose levels by triggering insulin secretion, delaying gastric emptying, and decreasing concentrations of glucagon in the plasma [32]. Being secreted by the L cells of the small intestine, this hormone is glucose-dependent. The increased secretion of insulin occurs via the incretin effect, in which oral glucose loads proportionally and stimulates its secretion. GLP agonists also reduce apoptosis of the pancreatic β-cells and, in fact, promote proliferation of them [32]. In a diabetic, this mechanism is defective [32]. Side effects of the GLP-1 agonists include nausea, vomiting, injection-site reactions, and hypoglycemia [32]. The medication is contraindicated in patients with a family history of medullary thyroid cancer or multiple endocrine neoplasia [32].

In this study, the participants were mainly prescribed 2.4 mg of semaglutide weekly and administered subcutaneously [32]. The FDA has approved semaglutide and high-dose liraglutide for the purpose of reduction in weight. The results of the 10 studies demonstrated that the GLP-1 receptor agonists, specifically semaglutide, aided in significant weight reductions compared to placebo [33]. Furthermore, GLP-1 agonists have a beneficial effect on the cardiovascular system by regulating endothelial function, blood pressure, and platelet function [34].

Gastrointestinal side effects were observed in the group receiving the semaglutide treatment vs placebo [33]. Discontinuation of treatment was also higher in the group receiving the medication [2]. This could be either due to the associated side effects or a discontinuation when the patient reduced a significant amount of weight. Discontinuation of treatment can easily lead to rebound weight gain. Another side effect of the treatment in obese individuals is the “Ozempic face,” in which the face sags due to the loss of volume since the adipose turnover is faster than the rate at which the skin underneath shrinks [34].

Strengths

This systematic review has many strengths and weaknesses. It follows the guidelines of PRISMA, which establishes an accurate and definite method to collect and extract data along with providing a quality assessment of the selected articles. The review incorporated a search strategy that extracted relevant articles from credible and distinguished databases. This study mainly focused on randomized controlled trials, which can establish causation in the most rigorous way and decrease selection or observer bias. Due to the large number of studies, the conclusions were clinically relevant. However, the comprehensive review examines the medical effects of GLP-1 receptor agonists in obese and overweight patients without diabetes. This is expected to provide a favorable method for obese patients along with lifestyle modifications. The study analyses the data and provides a thorough discussion regarding the pathophysiology of GLP-1 receptor agonists and their efficacy in not only reducing weight but also being a cardioprotective agent.

Limitations

The review also has its limitations. First, the FDA approved semaglutide and high-dose liraglutide for weight loss in 2021. Therefore, there are limited studies and not enough significant data to ultimately conclude that the results are clinically significant. Second, the small size of the study may alter the results and show no true differences. Third, there was a lack of certainty about whether the patients in this study had comorbid conditions such as heart failure or chronic kidney disease. Articles not written were excluded and this study only focused on publications within the past five to 10 years. Future research should also consider expanding the sample size of the studies. Finally, previous studies regarding semaglutide and weight reduction had been published, which affected the innovation of the current studies. This review focuses on the efficacy of a single drug. Future research should aim to focus on the mechanism and efficacy of dual drug therapy with GLP-1 agonists and gastric inhibitory polypeptide receptor (GIPR) agonists.

## Conclusions

GLP-1 receptor agonists have demonstrated efficacy in reducing weight in obese and overweight patients without diabetes. This review aimed to provide an up-to-date study for the overall body weight effects of GLP-1 receptor agonists in adults who were medically defined as obese or having a BMI greater than 27. Randomized controlled trials were the predominant studies assessed in this review. Compared to placebo, there was a significant reduction in weight (average of 6 kg) when the patient was treated with a GLP-1 agonist. Currently, two formulations of the medication semaglutide are available and safe. The first formulation Ozempic® is available in 0.5 mg or 1.0 mg. It is administered once weekly subcutaneously. Wegovy® is dosed at 2.4 mg. The second formulation, an oral semaglutide called Rybelsus® is prescribed for 7 g or 14 mg. With diet control and exercise interventions, semaglutide serves as a potent reducer of weight in obese individuals who look forward to improving their quality of life. The treatment demonstrated the greatest effect on losing body weight, maintaining lower glucose levels in the blood, and reducing blood pressure but it had many side effects as well. We hope that more trials conducted in the future can provide a clear picture and provide a strategy for clinical practice with regard to pharmacologic treatments of obesity.

## References

[1] Wilding JP, Batterham RL, Calanna S (2021). Once-weekly semaglutide in adults with overweight or obesity. N Engl J Med.

[2] Tan HC, Dampil OA, Marquez MM (2022). Efficacy and safety of semaglutide for weight loss in obesity without diabetes: a systematic review and meta-analysis. J ASEAN Fed Endocr Soc.

[3] Rubino DM, Greenway FL, Khalid U (2022). Effect of weekly subcutaneous semaglutide vs daily liraglutide on body weight in adults with overweight or obesity without diabetes: the STEP 8 randomized clinical trial. JAMA.

[4] O'Neil PM, Birkenfeld AL, McGowan B (2018). Efficacy and safety of semaglutide compared with liraglutide and placebo for weight loss in patients with obesity: a randomised, double-blind, placebo and active controlled, dose-ranging, phase 2 trial. Lancet.

[5] Garvey WT, Batterham RL, Bhatta M (2022). Two-year effects of semaglutide in adults with overweight or obesity: the STEP 5 trial. Nat Med.

[6] Ryan DH, Lingvay I, Colhoun HM (2020). Semaglutide effects on cardiovascular outcomes in people with overweight or obesity (select) rationale and design. Am Heart J.

[7] Ghusn W, De la Rosa A, Sacoto D (2022). Weight loss outcomes associated with semaglutide treatment for patients with overweight or obesity. JAMA Netw Open.

[8] Guo X, Zhou Z, Lyu X (2022). The antiobesity effect and safety of GLP-1 receptor agonist in overweight/obese patients without diabetes: a systematic review and meta-analysis. Horm Metab Res.

[9] Friedrichsen M, Breitschaft A, Tadayon S, Wizert A, Skovgaard D (2021). The effect of semaglutide 2.4 mg once weekly on energy intake, appetite, control of eating, and gastric emptying in adults with obesity. Diabetes Obes Metab.

[10] (2022). Adult obesity facts. https://www.cdc.gov/obesity/php/data-research/adult-obesity-facts.html?CDC_AAref_Val=https://www.cdc.gov/obesity/data/adult.html.

[11] Park Park, H H (2021). Overweight & obesity statistics. National Institute of Diabetes and Digestive and Kidney Diseases.

[12] Malik VS, Willett WC, Hu FB (2013). Global obesity: trends, risk factors and policy implications. Nat Rev Endocrinol.

[13] Papatheodorou K, Banach M, Bekiari E, Rizzo M, Edmonds M (2018). Complications of diabetes 2017. J Diabetes Res.

[14] Bailey CJ (2017). Metformin: historical overview. Diabetologia.

[15] Bergmann NC, Davies MJ, Lingvay I, Knop FK (2022). Semaglutide for the treatment of overweight and obesity: a review. Diabetes Obes Metab.

[16] Szanto KB, Li J, Cordero P, Oben JA (2019). Ethnic differences and heterogeneity in genetic and metabolic makeup contributing to nonalcoholic fatty liver disease. Diabetes Metab Syndr Obes.

[17] Lee HS, Lee J (2021). Effects of exercise interventions on weight, body mass index, lean body mass and accumulated visceral fat in overweight and obese individuals: a systematic review and meta-analysis of randomized controlled trials. Int J Environ Res Public Health.

[18] Heslehurst N, Vieira R, Akhter Z (2019). The association between maternal body mass index and child obesity: a systematic review and meta-analysis. PLoS Med.

[19] Simmonds M, Llewellyn A, Owen CG, Woolacott N (2016). Predicting adult obesity from childhood obesity: a systematic review and meta-analysis. Obes Rev.

[20] Safaei M, Sundararajan EA, Driss M, Boulila W, Shapi'i A (2021). A systematic literature review on obesity: understanding the causes & consequences of obesity and reviewing various machine learning approaches used to predict obesity. Comput Biol Med.

[21] Riaz H, Khan MS, Siddiqi TJ (2018). Association between obesity and cardiovascular outcomes: a systematic review and meta-analysis of Mendelian randomization studies. JAMA Netw Open.

[22] Tremmel M, Gerdtham UG, Nilsson PM, Saha S (2017). Economic burden of obesity: a systematic literature review. Int J Environ Res Public Health.

[23] Yang J, Hu J, Zhu C (2021). Obesity aggravates COVID-19: a systematic review and meta-analysis. J Med Virol.

[24] Chiavaroli L, Lee D, Ahmed A (2021). Effect of low glycaemic index or load dietary patterns on glycaemic control and cardiometabolic risk factors in diabetes: systematic review and meta-analysis of randomised controlled trials. BMJ.

[25] Rossboth S, Lechleitner M, Oberaigner W (2021). Risk factors for diabetic foot complications in type 2 diabetes - a systematic review. Endocrinol Diabetes Metab.

[26] Wulan SN, Westerterp KR, Plasqui G (2010). Ethnic differences in body composition and the associated metabolic profile: a comparative study between Asians and Caucasians. Maturitas.

[27] Galicia-Garcia U, Benito-Vicente A, Jebari S (2020). Pathophysiology of type 2 diabetes mellitus. Int J Mol Sci.

[28] Banday MZ, Sameer AS, Nissar S (2020). Pathophysiology of diabetes: an overview. Avicenna J Med.

[29] Rena G, Hardie DG, Pearson ER (2017). The mechanisms of action of metformin. Diabetologia.

[30] Pippitt K, Li M, Gurgle HE (2016). Diabetes mellitus: screening and diagnosis. Am Fam Physician.

[31] Latif W, Lambrinos KJ, Rodriguez R. (2024). Compare and contrast the glucagon-like peptide-1 receptor agonists (GLP1RAs). StatPearls [Internet].

[32] Tay JQ (2023). Ozempic face: a new challenge for facial plastic surgeons. J Plast Reconstr Aesthet Surg.

[33] Blundell J, Finlayson G, Axelsen M, Flint A, Gibbons C, Kvist T, Hjerpsted JB (2017). Effects of once-weekly semaglutide on appetite, energy intake, control of eating, food preference and body weight in subjects with obesity. Diabetes Obes Metab.

[34] Page MJ, McKenzie JE, Bossuyt PM (2021). The PRISMA 2020 statement: an updated guideline for reporting systematic reviews. Syst Rev.

